# MoTeX-II: structured MoTif eXtraction from large-scale datasets

**DOI:** 10.1186/1471-2105-15-235

**Published:** 2014-07-08

**Authors:** Solon P Pissis

**Affiliations:** 1 Department of Informatics, King’s College London, The Strand, WC2R 2LS London, UK

**Keywords:** Motif extraction, Structured motif, Transcription factor binding sites

## Abstract

**Background:**

Identifying repeated factors that occur in a string of letters or common factors that occur in a set of strings represents an important task in computer science and biology. Such patterns are called *motifs*, and the process of identifying them is called *motif extraction*. In biology, motif extraction constitutes a fundamental step in understanding regulation of gene expression. State-of-the-art tools for motif extraction have their own constraints. Most of these tools are only designed for *single* motif extraction; *structured* motifs additionally allow for distance intervals between their single motif components. Moreover, motif extraction from large-scale datasets—for instance, large-scale ChIP-Seq datasets—cannot be performed by current tools. Other constraints include high time and/or space complexity for identifying long motifs with higher error thresholds.

**Results:**

In this article, we introduce MoTeX-II, a word-based high-performance computing tool for structured MoTif eXtraction from large-scale datasets. Similar to its predecessor for single motif extraction, it uses state-of-the-art algorithms for solving the fixed-length approximate string matching problem. It produces similar and partially identical results to state-of-the-art tools for structured motif extraction with respect to accuracy as quantified by statistical significance measures. Moreover, we show that it matches or outperforms these tools in terms of runtime efficiency by merging single motif occurrences efficiently. MoTeX-II comes in three flavors: a standard CPU version; an OpenMP-based version; and an MPI-based version. For instance, the MPI-based version of MoTeX-II requires only a couple of hours to process all human genes for structured motif extraction on 1056 processors, while current sequential tools require more than a week for this task. Finally, we show that MoTeX-II is successful in extracting known composite transcription factor binding sites from real datasets.

**Conclusions:**

Use of MoTeX-II in biological frameworks may enable deriving reliable and important information since real full-length datasets can now be processed with almost *any* set of input parameters for both single and structured motif extraction in a reasonable amount of time. The open-source code of MoTeX-II is freely available at http://www.inf.kcl.ac.uk/research/projects/motex/.

## Background

Identifying repeated factors that occur in a string of letters or common factors that occur in a set of strings represents an important task in computer science and biology. Such patterns are called *motifs*, and the process of identifying them is called *motif extraction*. Motif extraction has numerous direct applications in areas that require some form of *text mining*, that is, the process of deriving reliable information from text [1]. Here we focus on its application to molecular biology.

In biological applications, motifs correspond to functional and/or conserved DNA, RNA, or protein sequences. Alternatively, they may correspond to (recently, in evolutionary terms) duplicated genomic regions, such as transposable elements or even whole genes. It is mandatory to allow for a certain number of errors between different occurrences of the same motif since both single nucleotide polymorphisms as well as errors introduced by wet-lab sequencing platforms might have occurred. Hence, molecules that encode the same or related functions do not necessarily have *exactly* identical sequences.

A *single* DNA motif is defined as a sequence of nucleic acids that has a specific biological function. The pattern can be fairly short, 5 to 20 base-pairs (bp) long, and is known to occur in different genes [2], or several times within the same gene [3]. The DNA motif extraction problem is the task of detecting overrepresented motifs as well as conserved motifs in a set of orthologous DNA sequences. Such conserved motifs may, for instance, be potential candidates for transcription factor binding sites for a regulatory protein [4].

In addition to this simple form of DNA motifs, structured motifs are another special type of DNA motifs. A *structured* DNA motif consists of two (or even more) smaller conserved sites separated by a *spacer* (gap). The spacer occurs in the middle of the motif because the transcription factors bind as a dimer. This means that the transcription factor is formed by two subunits having two separate contact points with the DNA sequence. These contact points are separated by a non-conserved spacer of mostly fixed or slightly variable length. Such conserved structured motifs may, for instance, be potential candidates for transcription factor binding sites for a composite regulatory protein [5].

In accordance with the pioneering work of Sagot *et al.*[6,7], we formally define the single and structured motif extraction problems as follows.

A *single motif* is a string of letters (word) on an alphabet *Σ*. Given an integer error threshold *e*, a motif on *Σ* is said to *e-occur* in a string *s* on *Σ*, if the motif and a factor (substring) of *s* differ by a (Hamming) distance of *e*. The *single motif extraction* problem takes as input a set *s*_1_,…,*s*_*N*_ of strings on *Σ*, where *N*≥2, the quorum 1≤*q*≤*N*, the maximal allowed distance *e* (error threshold), and the length *k* for the motifs. It consists in determining all motifs of length *k*, such that each motif *e*-occurs in at least *q* input strings. Such motifs are called *valid*.

A *structured motif* is a pair (*m*,*d*), where *m* = (*m*_*i*_)1≤*i*≤*β* is a *β*-tuple of single motifs, and $d={\left(d_{\min_{i}},d_{\max_{i}}\right)}_{1\leq i<\beta }$ is a *β*−1-tuple of pairs denoting *β*−1 intervals of distance between the *β* single motifs. A structured motif is denoted by

$$m_{1}\left[d_{\min_{1}},d_{\max_{1}}\right]m_{2}\ldots m_{\beta -1}\left[d_{\min_{\beta -1}},d_{\max_{\beta -1}}\right]m_{\beta }.$$

Each element *m*_*i*_ of a structured motif is called a *box* and its length is denoted by *k*_*i*_.

Given a *β*-tuple (*e*_*i*_)_1≤*i*≤*β*_ of error thresholds, a structured motif (*m*,*d*) is said to have an (*e*_*i*_)_1≤*i*≤*β*_-occurrence in a string *s* on *Σ* if, for all 1≤*i*≤*β*, there is an *e*_*i*_-occurrence *mi*′ of *m*_*i*_ such that: 

1. $m_{1}^{\prime },\ldots ,m_{\beta }^{\prime }$ are in *sand*

2. the distance between the end position of $m_{i}^{\prime }$ and the start position of $m_{i+1}^{\prime }$ in *s* is in $\left[d_{\min_{i}},d_{\max_{i}}\right]$, for all 1≤*i*<*β*.

The *structured motif extraction* problem takes as input a set *s*_1_,…,*s*_*N*_ of strings on *Σ*, where *N*≥2, the quorum 1≤*q*≤*N*, *β* lengths (*k*_*i*_)_1≤*i*≤*β*_, *β* error thresholds (*e*_*i*_)_1≤*i*≤*β*_, and *β*−1 intervals ${\left(d_{\min_{i}},d_{\max_{i}}\right)}_{1\leq i<\beta }$ of distance. Given these parameters, the problem consists in determining all structured motifs that have an (*e*_*i*_)_1≤*i*≤*β*_-occurrence in at least *q* input strings. Such structured motifs are called *valid*.

A problem instance is denoted by

$$\begin{array}{l} <\left(k_{1},e_{1}\right)\left[d_{\min_{1}},d_{\max_{1}}\right]\left(k_{2},e_{2}\right)\ldots \left(k_{\beta -1},e_{\beta -1}\right)\\ \;\left[d_{\min_{\beta -1}},d_{\max_{\beta -1}}\right]\left(k_{\beta },e_{\beta }\right),q>. \end{array}$$

### Related work

Most of the algorithms designed to find single and structured motifs use a set of promoter sequences of coregulated genes to identify statistically overrepresented motifs. In accordance with [8], the combinatorial approach used in their design leads to the following classification: 

1. *Word-based* methods that mostly rely on exhaustive enumeration, that is, counting and comparing oligonucleotide sequence (*k*-mer) frequencies;

2. *Probabilistic sequence* models, where the model parameters are estimated using maximum-likelihood or Bayesian inference methods.

Here we focus on word-based methods, since probabilistic sequence models often cannot converge to the global optimum. A plethora of word-based tools only for single motif extraction, such as YMF [9], Weeder [2], FLAME [10], and MoTeX[11] have already been released. In the search for more complex motifs, fewer methods have been released that extract DNA sites composed by two boxes, such as Dyad-Analysis [4] and MITRA [5]. To the best of our knowledge, there exist only two word-based tools that can address the problem for multiple boxes with distance intervals: RISOTTO [12] (the successor of RISO [7,13]) and EXMOTIF [14].

Let us first describe the approach used in RISOTTO for single motif extraction. This approach was first introduced by Sagot in [6]. RISOTTO initially indexes the set of *N* strings using a *truncated suffix tree*[15]. The suffix tree is then modified to store a boolean array of size *N* at each node of the suffix tree. This array indicates the strings in the input dataset that contain the factor labeling the path from the root to the corresponding tree node. RISOTTO subsequently searches for *e*-occurrences of motifs along different paths of the suffix tree. For every valid motif, one has to walk along at most *N*×*n* different paths in the suffix tree, where *n* is the average string length. For every string of length *k* induced by a path in the tree, there exist at most |*Σ*|^*e*^*k*^*e*^ valid motifs, where |*Σ*| is the size of the alphabet *Σ*, and *e* is the error threshold. Hence, the overall time complexity of this approach is $O\left(|\Sigma {|}^{e}k^{e}N^{2}n\right)$, where the additional factor *N* is required to access the boolean arrays.

For structured motif extraction, RISOTTO makes uses of an additional data structure, the *box-link*. This data structure is constructed to store the information needed to jump from box to box. Informally, a box-link is a tuple of tree nodes, corresponding to these jumps in the suffix tree. For clarity of description, let us assume that each box has the same length *k* and a fixed-length gap from the next box. The extraction of structured motifs starts by extracting single motifs of length *k*, one at a time. The suffix tree is temporarily and partially modified so as to extract the subsequent single motifs. When no errors are allowed, there exist at most |*Σ*|^*βk*^ ways of spelling all structured motifs. In this case, the total number of visits made to nodes between the root and level *k* of the suffix tree is bounded by $O\left(|\Sigma {|}^{\mathrm{\beta k}}\right)$. However, when up to *e* errors are allowed in each box, a node at level *k* may be visited $O\left(|\Sigma {|}^{\mathrm{\beta e}}k^{\mathrm{\beta e}}\right)$ times more; the total number of visits made to nodes between the root and level *k* of the suffix tree is $O\left(N|\Sigma {|}^{\beta (e+k)}k^{\mathrm{\beta e}}\right)$, where the additional factor *N* is required to access the boolean arrays. A number of operations is also needed to update and restore the suffix tree. In overall, the time complexity of RISOTTO for structured motif extraction is bounded by $O\left(N|\Sigma {|}^{\beta (e+k)}k^{\mathrm{\beta e}}\right)$.

EXMOTIF uses an inverted index of symbol positions, and it enumerates all structured motifs by *positional joins* over this index. The distance intervals constraints are also considered at the same time as the joins. Let us first describe the approach used in EXMOTIF for single motif extraction. There exist potentially |*Σ*|^*k*^ single motifs, and, therefore, in the worst case, $O\left(|\Sigma {|}^{k}\right)$ single motifs may be extracted. For a single motif of length *k*, EXMOTIF uses *O*(log*k*) positional joins to obtain the total number of input strings that contain at least one occurrence of the single motif, and each such join takes O(nN) time. Thus, extracting the single motifs takes time *O*(*nN* log(*k*)|*Σ*|^*k*^) in the worst case. For |*Σ*|^*k*^ single motifs, there exist |*Σ*|^*βk*^ potential structured motifs. When no errors are allowed, extracting the structured motifs requires time *O*(*βnN*|*Σ*|^*βk*^). However, when up to *e* errors are allowed in each box, extracting the structured motifs requires time *O*(*βnN*|*Σ*|^*βk*^+*β*^2^*k*^*e*^|*Σ*|^*e*^). Hence, in overall, the time complexity of EXMOTIF is bounded by *O*(*βnN*|*Σ*|^*βk*^+*nN* log(*k*)|*Σ*|^*k*^).

### Our contribution

All aforementioned algorithms for single and/or structured motif extraction exhibit all or a part of the following disadvantages: 

• Their time complexity depends on or grows exponentially with the motif length *k*. Hence, they can only be used for finding very short motifs [16]. For instance, YMF allows only up to *k*:=8 and Weeder up to *k*:=12.

• Their time complexity depends on the size |*Σ*| of the alphabet. Hence, they are not suitable for detecting motifs drawn from large alphabets (e.g., amino acids, where |*Σ*|=20).

• Their time complexity grows exponentially with the error threshold *e*. Thus, they are not suitable for detecting long motifs with higher error thresholds, say *k*:=13 and *e*:=4.

There are two additional disadvantages: 

• Existing tools are only designed for identifying motifs under the *Hamming distance* model (mismatches) but not under the *edit distance* model (indels). Indels in biological sequences may occur because of insertions or deletions of genomic segments at various genomic locations or due to sequencing errors.

• Existing tools are not designed or implemented for high-performance computing (HPC). For instance, Weeder and RISOTTO, which are currently two of the most widely used tools for motif extraction, require more than two months to process all human genes for single motif extraction, with *k*:=12 and *e*:=4, making this kind of analyses intractable [11]. A parallel algorithm for the extraction of structured motifs exists [17], but the implementation is not publicly maintained. Moreover, in [16], the authors mention that they plan to improve their algorithm’s ability to process large-scale ChIP-Seq datasets.

To alleviate these shortcomings, we have introduced MoTeX, a word-based HPC tool for single MoTif eXtraction [11]. A valid single motif is called *strictly valid* if it occurs *exactly* (with no errors), at least once, in any of the input strings. By making this stricter assumption for motif validity, we reduced the problem of single motif extraction in solving the *fixed-length approximate string matching* problem [18] for all *N*^2^ pairs of the *N* input strings. We demonstrated that this approach can alleviate *all* the aforementioned shortcomings of state-of-the-art tools for motif extraction; and produce very promising results both in terms of accuracy under statistical measures of significance as well as efficiency. A part of these well-known issues for single motif extraction were discussed and addressed in [19] and [20]. Notice that the reduction proposed here makes the time and space complexity of MoTeX not directly comparable to the ones of RISOTTO and EXMOTIF which solve a harder algorithmic problem.

In this article, since also most of the aforementioned tools are only designed for single motif extraction, we introduce MoTeX-II, the successor of MoTeX, for the more involved case of structured motif extraction from large-scale datasets. To detect the structured motifs, one may apply single motif extraction to detect each box separately. However, this solution breaks down when some boxes are insignificant. Thus, it is crucial to detect the whole structured motif directly whose spacers and other possibly significant boxes can increase its overall significance. Instead of computing a single dynamic-programming (DP) matrix for each pair of strings, we compute *β* DP matrices (one for each box); and then merge the single motif occurrences of the individual boxes using the intervals of distance to determine whether they form a valid structured motif or not.

MoTeX-II produces similar and partially identical results to current state-of-the-art tools for structured motif extraction with respect to accuracy as quantified by statistical significance measures. Moreover, we show that it matches or outperforms these tools in terms of runtime efficiency by merging single motif occurrences efficiently. MoTeX-II comes in three flavors: a standard CPU version; an OpenMP-based version; and an MPI-based version. For instance, the MPI-based version of MoTeX-II requires only a couple of hours to process all human genes for structured motif extraction on 1056 processors, while current sequential tools require more than a week for this task. Finally, we show that MoTeX-II is successful in extracting known composite transcription factor binding sites from real datasets.

## Methods

### Definitions and notation

In this section, in order to provide an overview of the algorithms used later on, we give a few definitions, generally following a standard textbook of algorithms on strings [21].

An *alphabetΣ* is a finite non-empty set whose elements are called *letters*. A *string* on an alphabet *Σ* is a finite, possibly empty, sequence of elements of *Σ*. The zero-letter sequence is called the *empty string*, and is denoted by *ε*. The *length* of a string *x* is defined as the length of the sequence associated with the string *x*, and is denoted by |*x*|. We denote by *x*[*i*], for all 1≤*i*≤|*x*|, the letter at index *i* of *x*. Each index *i*, for all 1≤*i*≤|*x*|, is a position in *x* when *x*≠*ε*. It follows that the *i*th letter of *x* is the letter at position *i* in *x*, and that *x*=*x*[1.. |*x*|].

A string *x* is a *factor* of a string *y* if there exist two strings *u* and *v*, such that *y*=*uxv*. Let the strings *x*,*y*,*u*, and *v*, such that *y*=*uxv*. If *u*=*ε*, then *x* is a *prefix* of *y*. If *v*=*ε*, then *x* is a *suffix* of *y*.

Let *x* be a non-empty string and *y* be a string. We say that there exists an (exact) *occurrence* of *x* in *y*, or, more simply, that *xoccurs* (exactly) in *y*, when *x* is a factor of *y*. Every occurrence of *x* can be characterised by a position in *y*. Thus we say that *x* occurs at the *starting positioni* in *y* when *y*[*i*.. *i*+|*x*|−1]=*x*. It is sometimes more suitable to consider the *ending positioni*+|*x*|−1.

The *edit distance*, denoted by *δ*_*E*_(*x*,*y*), for two strings *x* and *y* is defined as the minimum total cost of operations required to transform string *x* into string *y*. For simplicity, we only count the number of edit operations and consider that the cost of each edit operation is 1. The allowed operations are the following: 

• Ins: insert a letter in *y*, not present in *x*; (*ε*,*b*), *b*≠*ε*;

• Del: delete a letter in *y*, present in *x*; (*a*,*ε*), *a*≠*ε*;

• Sub: substitute a letter in *y* with a letter in *x*; (*a*,*b*), *a*≠*b*, *a*,*b*≠*ε*.

The Hamming distance *δ*_*H*_ is only defined on strings of the same length. For two strings *x* and *y*, *δ*_*H*_(*x*,*y*) is the number of positions in which the two strings differ, that is, have different letters. For the sake of completeness, we define *δ*_*H*_(*x*,*y*)=*∞* for strings *x*, *y* such that |*x*|≠|*y*|.

### Algorithms

In this section, we first formally define the *fixed-length approximate string matching* problem under the edit distance model and under the Hamming distance model; and provide a brief description and analysis of the algorithms to solve it. We show how the structured motif extraction problem can be reduced to the fixed-length approximate string matching problem, by using a stricter assumption than the one in the initial problem definition for the validity of structured motifs. Then, we provide an informal structure of our approach. Finally, we present a practical improvement on this approach by merging single motif occurrences efficiently.

#### Problem 1 (Edit distance)

Given a string *x* of length *m*, a string *y* of length *n*, an integer *k*, and an integer *e*<*k*, find all factors of *y*, which are at an edit distance less than, or equal to, *e* from every factor of fixed length *k* of *x*.

#### Problem 2 (Hamming distance)

Given a string *x* of length *m*, a string *y* of length *n*, an integer *k*, and an integer *e*<*k*, find all factors of *y*, which are at a Hamming distance distance less than, or equal to, *e* from every factor of fixed length *k* of *x*.

Let D [0.. *n*,0.. *m*] be a DP matrix, where D [*i*,*j*] contains the edit distance between some factor *y*[*i*^′^.. *i*] of *y*, for some 1≤*i*^′^≤*i*, and factor *x*[max{1,*j*−*k*+1}.. *j*] of *x*, for all 1≤*i*≤*n*, 1≤*j*≤*m*. This matrix can be obtained through a straightforward O(kmn)-time algorithm by constructing DP matrices D^*s*^[0.. *n*,0.. *k*], for all 1≤*s*≤*m*−*k*+1, where D^*s*^[*i*,*j*] is the edit distance between some factor of *y* ending at *y*[*i*] and the prefix of length *j* of *x*[*s*.. *s*+*k*−1]. We obtain D by collating D^1^ and the last row of D^*s*^, for all 2≤*s*≤*m*−*k*+1. We say that *x*[max{1,*j*−*k*+1}.. *j*]*e*-occurs in *y* ending at *y*[*i*]*iff* D[*i*,*j*]≤*e*, for all 1≤*j*≤*m*, 1≤*i*≤*n*.

Iliopoulos, Mouchard, and Pinzon devised MaxShift[18], an algorithm with time complexity O(m⌈k/w⌉n), where *w* is the size of the computer word. By using word-level parallelism, MaxShift can compute matrix D efficiently. The algorithm requires constant time for computing each cell D[*i*,*j*] by using word-level operations, assuming that *k*≤*w*. In the general case, it requires O(⌈k/w⌉) time. Hence, algorithm MaxShift requires time O(mn), under the assumption that *k*≤*w*. Notice that the space complexity is only O(m) since each row of D only depends on the immediately preceding row.

#### Theorem 1 ([18])

Given a string *x* of length *m*, a string *y* of length *n*, an integer *k*, and the size of the computer word *w*, matrix D can be computed in time O(m⌈k/w⌉n).

Let M [0.. *n*,0.. *m*] be a DP matrix, where M[*i*,*j*] contains the Hamming distance between factor *y*[max{1,*i*−*k*+1}.. *i*] of *y* and factor *x*[max{1,*j*−*k*+1}.. *j*] of *x*, for all 1≤*i*≤*n*, 1≤*j*≤*m*. Crochemore, Iliopoulos, and Pissis devised an analogous algorithm [22] that solves the analogous problem under the Hamming distance model with the same time and space complexity.

#### Theorem 2 ([22])

Given a string *x* of length *m*, a string *y* of length *n*, an integer *k*, and the size of the computer word *w*, matrix M can be computed in time O(m⌈k/w⌉n).

On the one hand, if the input dataset is relatively large, the possibility that there exists a structured motif which does not occur exactly, at least once, in the dataset *and* it also satisfies *all* the restrictions imposed by the input parameters, is rather unlikely, from both a combinatorial and a biological point of view. On the other hand, if the input dataset is rather small, single and structured motif extraction could potentially be performed by applying multiple sequence alignment to the input strings or exhaustive enumeration. We are therefore able to make the following stricter assumption for the validity of structured motifs.

#### Definition 1

A valid structured motif is called *strictly valid* if it occurs *exactly*, at least once, in any of the input strings.

Assuming that *k*≤*w*, the single motif extraction problem for strictly valid motifs can be solved in time $O(n^{2})$ per DP matrix, where *n* is the average length of the *N* strings, thus $O(N^{2}n^{2})$ in total [11]. For structured motif extraction, instead of computing a single DP matrix for each pair of strings, we compute *β* DP matrices (one for each box), and then merge the single motif occurrences of the individual boxes using the intervals of distance to determine whether they form a valid structured motif or not. For each pair of input strings, the DP-matrices computation requires time $O\left(\beta n^{2}\right)$. For a pair *x* and *y* of input strings, assume the value of a cell of the first DP matrix is less than or equal to *e*_1_, denoting an *e*_1_-occurrence of box *m*_1_ in *y*. Further, let $\delta :=\max\left\{d_{\max_{i}}-d_{\min_{i}}+1:1\leq i<\beta \right\}$ and *γ*:=*β*−1. For an (*e*_*i*_)_1≤*i*≤*β*_-occurrence of a structured motif in *y*, there exist $O(\delta^{\gamma })$ possible distance sequences, each of length *γ*. Merging the elements of these distance sequences for *x* and *y*, for each interval separately, in a *trivial* way gives $O\left(\gamma \delta^{2\gamma }\right)$ cells we have to check; thus, $O\left(\gamma \delta^{2\gamma }n^{2}\right)$, in total. Combined with the time for the DP-matrices computation, in overall, the algorithm requires time $O\left(N^{2}\left(\beta +\gamma \delta^{2\gamma }\right)n^{2}\right)=O\left(N^{2}\beta \delta^{2\gamma }n^{2}\right)$. In the case when each box has a fixed-length gap from the next box, that is, *δ*=1, the algorithm requires time $O\left(N^{2}\beta n^{2}\right)$.

#### Example 1

Let the input strings CAAACCTTT and CGAAAGTAT, and the problem instance <(3,0)[1,2](3,1),2> under the Hamming distance model. The algorithm starts by computing the DP matrix M for *x*:=CAAACCTTT, *y*:=CGAAAGTAT, and *k*_1_=*k*_2_:=3. 

After the DP-matrix computation, the algorithm continues by looking for *i*,*j*≥*k*_1_, such that M [*i*,*j*]≤*e*_1_. The algorithm finds M [5,4]=0≤*e*_1_, since *δ*_*H*_(*x*[2.. 4],*y*[3.. 5])=0. There exist *δ*^*γ*^=2 possible distance sequences, *s*_1_=1 and *s*_2_=2, each of length 1. Let *i*^′^=:*i*+*k*_1_=8 and *j*^′^=:*j*+*k*_1_=7. In order to merge the elements of sequences *s*_1_ and *s*_2_ for a potential *e*_2_−occurrence of the second box, we have to check the value of *δ*^2*γ*^=4 cells: M [*i*^′^+1,*j*^′^+1]; M [*i*^′^+1,*j*^′^+2]; M [*i*^′^+2,*j*^′^+1]; and M [*i*^′^+2,*j*^′^+2]. Only cell M [*i*^′^+1,*j*^′^+2]=M [9,9]=1≤*e*_2_, since *δ*_*H*_(*x*[7.. 9],*y*[7.. 9])=1. Since *q*=2, AAA[1,2]TTT is a valid structured motif occurring in both CAAACCTTT and CGAAAGTAT. The algorithm continues by computing the DP matrix for *x*:=CGAAAGTAT, *y*:=CAAACCTTT, and *k*_1_=*k*_2_:=3. 

After the DP-matrix computation, the algorithm continues by looking for *i*,*j*≥*k*_1_, such that M [*i*,*j*]≤*e*_1_. The algorithm finds M [4,5]=0≤*e*_1_, since *δ*_*H*_(*x*[3.. 5],*y*[2.. 4])=0. Let *i*^′^=:*i*+*k*_1_=7 and *j*^′^=:*j*+*k*_1_=8. In order to merge the elements of sequences *s*_1_ and *s*_2_ for a potential *e*_2_-occurrence of the second box, we have to check the value of *δ*^2*γ*^=4 cells: M [*i*^′^+1,*j*^′^+1]; M [*i*^′^+1,*j*^′^+2]; M [*i*^′^+2,*j*^′^+1]; and M [*i*^′^+2,*j*^′^+2]. Only cell M [*i*^′^+2,*j*^′^+1]=M [9,9]=1≤*e*_2_, since *δ*_*H*_(*x*[7.. 9],*y*[7.. 9])=1. Since *q*=2, AAA[1,2]TAT is a valid structured motif occurring in both CAAACCTTT and CGAAAGTAT.

A practical improvement on the runtime of the proposed algorithm can be achieved by the following observation, presented also, within a different context, in [7,13]. The cumulative distance between two boxes distanced by $d_{\min_{i}}$, from box *m*_*i*_ to box *m*_*i*+1_, and $d_{\min_{i+1}}+1$, from box *m*_*i*+1_ to box *m*_*i*+2_, is equivalent, from box *m*_*i*+2_ on, to the distance between boxes distanced by $d_{\min_{i}}+1$, from box *m*_*i*_ to box *m*_*i*+1_, and $d_{\min_{i+1}}$, from box *m*_*i*+1_ to box *m*_*i*+2_. In other words, it holds that $d_{\min_{i}}+\left(d_{\min_{i+1}}+1\right)=\left(d_{\min_{i}}+1\right)+d_{\min_{i+1}}$. Based on this fact, limited to the *i*th distance interval, the *prefix sums* of these distance sequences form a finite arithmetic progression $d_{\min_{1}}+\cdots +d_{\min_{i}},\ldots ,d_{\max_{1}}+\cdots +d_{\max_{i}}$ of length O(δγ). Assume the value of a cell of the first DP matrix is less than or equal to *e*_1_, denoting an *e*_1_-occurrence of box *m*_1_. Merging the elements of these progressions for each interval separately gives only $O(\gamma {\left(\delta \gamma \right)}^{2})$=$O\left(\delta^{2}\gamma^{3}\right)$ cells we have to check. Since the information for potential *e*_*i*_-occurrences of box *m*_*i*_, for all 2≤*i*≤*β*, is stored in the DP matrices, we may invalidate some *c*>0 of the $O\left(\delta^{2\gamma }\right)$ candidates that can never yield an (*e*_*i*_)_1≤*i*≤*β*_-occurrence in time $O\left(\delta^{2}\gamma^{3}+c\right)$ per *e*_1_-occurrence. Notice that these arithmetic progressions, and, hence, the association of the corresponding boxes with the candidates, can be precomputed, only once, since they are independent of the pairs of strings. Thus, in practice, we may avoid the enumeration of all $O\left(\gamma \delta^{2\gamma }\right)$ DP-matrix cells. However, in the worst case, the overall time complexity of the proposed algorithm remains $O\left(N^{2}\beta \delta^{2\gamma }n^{2}\right)$.

#### Example 2

Let the structured motif *m*_1_[1,2]*m*_2_[4,5]*m*_3_, where *k*_1_=*k*_2_=*k*_3_. The arithmetic progression for the first distance interval is given by $p_{1}:=d_{\min_{1}},\ldots ,d_{\max_{1}}$, that is *p*_1_=1,2; and for the second by $p_{2}:=d_{\min_{1}}+d_{\min_{2}},\ldots ,d_{\max_{1}}+d_{\max_{2}}$, that is *p*_2_=5,6,7. Therefore by considering only |*p*_1_|^2^+|*p*_2_|^2^=13 DP-matrix cells, we may invalidate some of the *δ*^2*γ*^=16 candidates that can never yield an (*e*_*i*_)_1≤*i*≤3_-occurrence. Thus, we may avoid enumerating all *γδ*^2*γ*^=32 cells. This is due to the fact that this enumeration consists of only 13 distinct cells. For instance, assume M [*i*,*j*]≤*e*_1_, denoting an *e*_1_-occurrence of box *m*_1_. Let *i*^′^=:*i*+*k*_1_ and *j*^′^=:*j*+*k*_1_. If cell M [*i*^′^+2,*j*^′^+1]>*e*_2_, then we can invalidate 4 candidates. This is because the association of this cell with the 4 candidates can be precomputed.

## Results

All experiments were conducted on an Infiniband-connected cluster using 1 up to 1056 cores of Intel Xeon Processors E5645 at 2.4 GHz running GNU/Linux. All programmes were compiled with gcc version 4.6.3 at optimisation level 3 (−O3). For clarity, in the rest of this section, a problem instance is denoted by

$$\begin{array}{l} <\left(k_{1},e_{1}\right)\left[d_{\min_{1}},d_{\max_{1}}\right]\left(k_{2},e_{2}\right)\ldots \left(k_{\beta -1},e_{\beta -1}\right)\\ \;\left[d_{\min_{\beta -1}},d_{\max_{\beta -1}}\right]\left(k_{\beta },e_{\beta }\right),q^{\prime }>, \end{array}$$

where *q*^′^ is the ratio (%) of *q* to *N*.

### Implementation

MoTeX-II was implemented in the C programming language under GNU/Linux. We implemented MoTeX-II in three flavors: a standard CPU version; an OpenMP version; and an MPI version. The parallelisation scheme is beyond the scope of this article; it can be found in [11]. SMILE [23] may be used as a post-analysis programme that, given the output of a motif extractor and the input dataset, calculates the *z*-score and other statistical measures for assessing the statistical significance of the reported motifs. The significance of the reported motifs is computed from their occurrence frequency in a random subset of the input dataset. The *support* of a reported motif is defined as the total number of input sequences that contain at least one occurrence of the reported motif. The *weighted support* is defined as the total number of occurrences of the reported motif over all input sequences. Given the support and weighted support for each reported motif in the input dataset, SMILE computes two *z*-scores based on the corresponding support and weighted support in the random subset. Finally, SMILE sorts the motifs by their *z*-scores in descending order, thereby providing two ranks for each reported motif. MoTeX-II can produce a SMILE-compatible output file, which can then directly be used as input for SMILE. MoTeX-II is distributed under the GNU General Public License (GPL). The open-source code, the documentation, and all of the datasets referred to in this section are publicly maintained at http://www.inf.kcl.ac.uk/research/projects/motex/.

### Accuracy

Although MoTeX-II is based on an exact and deterministic algorithm, we initially evaluated its accuracy. The reason for doing this is twofold: first, to ensure that our implementation is correct; and, second, to evaluate the impact of our stricter motif validity assumption (Definition 1). In accordance with the work of Buhler and Tompa [24], the testing samples were generated synthetically using the following steps: 

1. *β* single motifs *m*_1_,…,*m*_*β*_ of lengths *k*_1_,…,*k*_*β*_, respectively, were generated by randomly picking *k*_1_+⋯+*k*_*β*_ letters from the DNA alphabet *Σ*:={A,C,G,T}.

2. As basic input dataset, we used *N*=1,062 upstream sequences of *Bacillus subtilis* genes of total size 240 KB, obtained from the GenBank [25] database (see [23], for details).

3. *q* (*q*≤*N*) sequences were randomly selected from these *N* background sequences.

4. The following steps were performed for each of the *q* selected background sequences: 

(a) An instance $m_{i}^{\prime }$, for all 1≤*i*≤*β*, of the single motif *m*_*i*_ was obtained by randomly choosing *e*_*i*_ (*e*_*i*_<*k*_*i*_) positions and randomly replacing these *e*_*i*_ letters to one of the four letters in *Σ*.

(b) *γ*:=*β*−1 factors (spacers) *g*_1_,…,*g*_*γ*_ of lengths *d*_1_,…,*d*_*γ*_, respectively, were randomly generated by randomly picking $d_{1}+\cdots +d_{\gamma }\left(d_{\min_{1}}\;\leq d_{1}\;\leq d_{\max_{1}},\ldots ,d_{\min_{\gamma }}\;\leq d_{\gamma }\;\leq d_{\max_{\gamma }}\;\right)$ letters from *Σ*.

(c) An instance $m^{\prime }:=m_{1}^{\prime }g_{1}m_{2}^{\prime }g_{2}\ldots g_{\gamma }m_{\beta }^{\prime }$ of the structured motif was generated.

(d) A factor *r* of length *k*_1_+*d*_1_+⋯+*d*_*γ*_+*k*_*β*_ was randomly selected from the background sequence.

(e) Factor *r* was replaced by the generated instance *m*^′^ of the structured motif.

By following these steps, we implanted 100 motifs in the basic dataset for different combinations of input parameters. The results in Table 1 demonstrate the high accuracy of MoTeX-II. It was always able to identify all implanted motifs. We repeated the same experiment by implanting a single motif in the basic dataset for different combinations of input parameters to evaluate the accuracy of MoTeX-II under statistical measures of significance using SMILE. The results in Table 2 confirm the high accuracy of MoTeX-II. It was always able to identify the implanted motif with the *highest* rank. We also make available, on the website of MoTeX-II, the open-source code, the documentation, and the basic input dataset used to generate the aforementioned synthetic datasets for reproducing the results in Tables 1 and 2.

**Table 1:** **Number of motifs identified by **MoTeX-II** using a synthetic dataset**

**Parameters**	**Implanted**	**Identified**	**Extracted**
	**motifs**	**implanted motifs**	**motifs**
<(8,1)[3,3](8,1),7>	100	100	100
<(8,1)[3,3](8,1),15>	100	100	105
<(8,1)[3,3](9,2),7>	100	100	100
<(8,1)[3,3](9,2),15>	100	100	100
<(9,2)[3,3](8,1),7>	100	100	128
<(9,2)[3,3](8,1),15>	100	100	120
<(9,2)[3,3](9,2),7>	100	100	101
<(9,2)[3,3](9,2),15>	100	100	100

The number of motifs identified by MoTeX-II using a synthetic dataset. The basic input dataset consists of 1,062 upstream sequences of *Bacillus subtilis* genes of total size 240 KB.

**Table 2:** **Statistical evaluation of motifs identified by **MoTeX-II** using a synthetic dataset**

**Parameters**	**Implanted**	**Identified**	**Extracted**	**Ranking of**
	**motifs**	**implanted motifs**	**motifs**	**implanted motif**
<(3,0)[2,2](5,0),7>	1	1	5	1/1
<(5,0)[2,2](3,0),7>	1	1	6	1/1
<(3,0)[2,2](6,1),7>	1	1	2,475	1/1
<(6,1)[2,2](3,0),7>	1	1	2,753	1/1
<(5,1)[2,2](6,1),7>	1	1	17,118	1/1
<(6,1)[2,2](5,1),7>	1	1	17,135	1/1

*Ranking* stands for the *z*-score ranking of the identified implanted motif based on support/weighted support.

The statistical evaluation of the motifs identified by MoTeX-II using a synthetic dataset. The basic input dataset consists of 1,062 upstream sequences of *Bacillus subtilis* genes of total size 240 KB.

### Efficiency

To evaluate the efficiency of MoTeX-II, we compared its performance to the corresponding performance of RISOTTO and EXMOTIF, which are currently the most widely-used tools for structured motif extraction.

First, we compared the standard CPU version and the OpenMP-based version of MoTeX-II against RISOTTO and EXMOTIF for the structured motif extraction problem using a small-scale dataset. As input dataset, we used 250 randomly selected 1,000 bp-long upstream sequences of *Homo sapiens* genes with a total size of 250 KB, retrieved from the ENSEMBL [26] database. We used the −1,000 to −1 upstream regions. We measured the elapsed time for each programme for different combinations of input parameters. In particular, we provided different values for the single motif lengths *k*_1_,*k*_2_, the error thresholds *e*_1_,*e*_2_, and the quorum *q*^′^. As depicted in Table 3, the performance of MoTeX-II is *independent* of the aforementioned input parameters and corroborates our theoretical findings. The standard CPU version of MoTeX-II is competitive for short motifs and becomes the fastest as the lengths *k*_1_,*k*_2_ for the motifs and the error thresholds *e*_1_,*e*_2_ increase. As expected, the OpenMP-based version of MoTeX-II with 48 processing threads (-t 48) is always the fastest.

**Table 3:** **Elapsed-time comparison of RISOTTO, EXMOTIF, and **MoTeX-II** using a small-scale real dataset**

**Parameters**	**RISOTTO**	**EXMOTIF**	MoTeX-II-CPU	MoTeX-II-OMP-t 48
<(8,1)[2,3](8,1),7>	286s	898s	1,885s	46s
<(8,1)[2,3](8,1),15>	217s	626s	1,860s	48s
<(8,1)[2,3](9,2),7>	2,086s	2,253s	1,871s	49s
<(8,1)[2,3](9,2),15>	1,103s	2,222s	1,860s	48s
<(9,2)[2,3](8,1),7>	4,868s	2,222s	1,868s	48s
<(9,2)[2,3](8,1),15>	4,279s	2,197s	1,856s	49s
<(9,2)[2,3](9,2),7>	39,488s	22,862s	1,871s	47s
<(9,2)[2,3](9,2),15>	21,274s	22,739s	1,865s	47s

Elapsed-time comparison of RISOTTO, EXMOTIF, and MoTeX-II using a small-scale real dataset. The input dataset consists of 250 upstream sequences of *Homo sapiens* genes of total size 250 KB.

Then, we compared the OpenMP-based version of MoTeX-II against RISOTTO and EXMOTIF for the structured motif extraction problem using a medium-scale dataset. As input dataset, we used the full upstream *Yeast* genes dataset obtained from the GenBank database. We used the −1,000 to −1 upstream regions, truncating the region if and where it overlaps with an upstream open-reading frame (ORF). The input dataset consists of 5,796 upstream sequences of total size 3.7 MB. We measured the elapsed time for each programme for different combinations of input parameters. As depicted in Table 4, the performance of MoTeX-II is independent of the aforementioned input parameters. The OpenMP-based version of MoTeX-II finishes each assignment in a reasonable amount of time (2 hours), as opposed to RISOTTO, which requires more than a week for some assignments, and EXMOTIF, which is terminated by a segmentation fault. Notice that for most of the problem instances in Table 4, the OpenMP-based version of MoTeX-II with 48 processing threads accelerates the computations by more than a factor of 48 compared to RISOTTO, implying that the CPU version of MoTeX-II is also faster.

**Table 4:** **Elapsed-time comparison of RISOTTO, EXMOTIF, and **MoTeX-II** using a medium-scale real dataset**

**Parameters**	**RISOTTO**	**EXMOTIF**	MoTeX-II-OMP
			-t 48
<(8,1)[3,5](8,1),10>	1,015s	**	6,853s
<(8,1)[3,5](8,1),20>	423s	**	6,848s
<(8,1)[3,5](10,3),10>	*	**	6,865s
<(8,1)[3,5](10,3),20>	41,310s	**	6,915s
<(10,3)[3,5](8,1),10>	492,282s	**	7,002s
<(10,3)[3,5](8,1),20>	*	**	6,976s
<(10,3)[3,5](10,3),10>	*	**	7,008s
<(10,3)[3,5](10,3),20>	*	**	7,005s

^*^The programme did not terminate after one week of execution.

^**^The programme was terminated by a segmentation fault.

Elapsed-time comparison of RISOTTO, EXMOTIF, and MoTeX-II using the full upstream *Yeast* genes dataset. The input dataset consists of 5,796 upstream sequences of total size 3.7 MB.

Finally, we compared the MPI-based version of MoTeX-II against RISOTTO and EXMOTIF for the structured motif extraction problem using a large-scale dataset. As input dataset, we used the full upstream *Homo sapiens* genes dataset obtained from the ENSEMBL database. We used the −1,000 to −1 upstream regions. The input dataset consists of 19,535 upstream sequences of total size 22.2 MB. We measured the elapsed time for each programme for different combinations of input parameters. Although a direct comparison between the MPI-based version of MoTeX-II, RISOTTO, and EXMOTIF is unfair, we believe that it is critical as it highlights the fact that real full-length datasets cannot be processed by state-of-the-art tools for structured motif extraction in a reasonable amount of time; in other words, the time-to-solution *is* an important property. As depicted in Table 5, the MPI-based version of MoTeX-II with 1056 processors (-np 1056) finishes each assignment in a reasonable amount of time (2-3 hours), as opposed to RISOTTO and EXMOTIF, which require more than a week.

**Table 5:** **Elapsed-time comparison of RISOTTO, EXMOTIF, and **MoTeX-II** using a large-scale real dataset**

**Parameters**	**RISOTTO**	**EXMOTIF**	MoTeX-II-MPI-np 1056
<(8,1)[2,3](9,2)[3,5](10,3),5>	*	*	12,068s
<(8,1)[2,3](10,3)[3,5](9,2),5>	*	*	12,371s
<(9,2)[2,3](8,1)[3,5](10,3),5>	*	*	11,953s
<(9,2)[2,3](10,3)[3,5](8,1),5>	*	*	12,095s
<(10,3)[2,3](8,1)[3,5](9,2),5>	*	*	12,035s
<(10,3)[2,3](9,2)[3,5](8,1),5>	*	*	11,729s

^*^The programme did not terminate after one week of execution.

Elapsed-time comparison of RISOTTO, EXMOTIF, and MoTeX-II using the full upstream *Homo Sapiens* genes dataset. The input dataset consists of 19,535 upstream sequences of total size 22.2 MB.

### Real applications

To further evaluate the accuracy of MoTeX-II in extracting known composite transcription factor binding sites from real datasets, we compared its output to the corresponding output of EXMOTIF using SMILE.

**Application I:** In accordance with [14], we evaluated the accuracy of MoTeX-II by extracting the conserved features of known transcription factor binding sites in *Yeast*. In particular, we used the binding sites for the Zinc (Zn) factors [27]. There exist 11 binding sites listed for the Zn cluster, 3 of which are single motifs. The remaining 8 are structured, as shown in Table 6. For the evaluation, we first formed several problem instances according to the conserved features in the binding sites. Then we extracted the valid structured motifs satisfying these parameters from the upstream regions of 68 genes regulated by Zn factors [27]. We used the −1,000 to −1 upstream regions, truncating the region if and where it overlaps with an upstream ORF. After extraction, since binding sites cannot have many occurrences in the ORF regions—in the genes—we excluded some motifs if they are also valid in the ORF regions. Finally, we computed the *z*-scores for the remaining valid motifs, and ranked them by descending *z*-scores using SMILE. We set *q*^′^=7 within the upstream regions and *q*^′^=30 within the ORF regions, empirically determined in [14]. As shown in Table 6, we can successfully predict GAL4, GAL4 chips, LEU3, PPR1, and PUT3 with the *highest* rank. CAT8, HAP1, and LYS also have high ranks. We were thus able to extract all 8 transcription factors for the Zn factors with high confidence. As a direct comparison, similar and partially identical results were reported by EXMOTIF (see Table 6). The small differences observed in Table 6 between ranks of the highest scoring motifs reported by the two programmes are due to the randomisation in SMILE. Notice that the final (original) number of motifs extracted (original is before excluding the motifs that are also valid in the ORF regions) is identical; showing that our stricter assumption for motif validity is also reasonable with real datasets.

**Table 6:** **Extraction of transcription factors for the Zinc factors by EXMOTIF and **MoTeX-II

			**EXMOTIF**		MoTeX-II	
**TF name**	**Known motif**	**Predicted Motif**	**Extracted motifs**	**Ranking**	**Extracted motifs**	**Ranking**
GAL4						
GAL4 chips	CGGRnnRCYnYnCnCCG	CGG[11,11]CCG	1634(3346)	1/1	1634(3346)	1/1
CAT8	CGGnnnnnnGGA	CGG[6,6]GGA	1621(3356)	451/73	1621(3356)	359/51
HAP1	CGGnnnTAnCGGCGGnnnTAnCGGnnnTA	CGG[6,6]CGG	1621(3356)	84/96	1621(3356)	73/85
LEU3	RCCGGnnCCGGY	CCG[4,4]CGG	1588(3366)	2/2	1588(3366)	1/2
LYS	WWWTCCRnYGGAWWW	TCC[3,3]GGA	1605(3360)	39/25	1605(3360)	32/17
PPR1	WYCGGnnWWYKCCGAW	CGG[6,6]CCG	1621(3356)	1/2	1621(3356)	1/2
PUT3	YCGGnAnGCGnAnnnCCGA					
	CGGnAnGCnAnnnCCGA	CGG[10,11]CCG	727(4035)	1/1	727(4035)	1/1

*TF name* stands for transcription factor name; *Known Motif* stands for the known binding sites corresponding to the transcription factors in TF name column; *Predicted Motif* stands for the motifs extracted by EXMOTIF and MoTeX-II, respectively; *Extracted motifs* gives the final (original) number of motifs extracted (original is before excluding the motifs that are also valid in the ORF regions); *Ranking* stands for the *z*-score ranking based on support/weighted support.

The extraction of transcription factors for the Zinc factors by EXMOTIF and MoTeX-II.

**Application II:** The complex transcriptional regulatory network in Eukaryotic organisms usually requires interactions of multiple transcription factors. A potential application of MoTeX-II is to extract such composite regulatory binding sites from DNA sequences. In accordance with [14], we considered two such transcription factors, URS1H and UASH, which are involved in early meiotic expression during sporulation, and that are known to coregulate 11 *Yeast* genes [28]. These 11 genes are also listed in SCPD [29], the promoter database of *Saccharomyces cerevisiae*. In 10 of those genes the URS1H binding site appears downstream from UASH; in the remaining one (HOP1) the binding sites are reversed. We applied multiple sequence alignment to the 10 genes (all except HOP1); and then obtained their consensus:

$$\text{taTTTtGGAGTaata}\;\left[4,179\right]\;\text{ttGGCGGCTAA}.$$

The lower-case letters are less conserved, whereas the upper-case letters are the most conserved. Based on the most conserved factors of the consensus and the parameters empirically determined in [14], we formed the following problem instance:

$$<(3,1)\left[1,1\right](5,2)\left[10,185\right](9,1),70>.$$

Notice that the distance of length 6 added to the interval [4,179] is to account for the non-conserved positions. We then extracted the structured motifs in the upstream regions of the 10 genes. We used the −800 to −1 upstream regions, and truncated the segment if it overlaps with an upstream ORF. We set *q*^′^=10 within the ORF regions, also empirically determined in [14]. MoTeX-II was able to identify the real motif

$$\text{TTT}\;\left[1,1\right]\;\text{GGAGT}\;\left[10,185\right]\;\text{GGCGGCTAA}$$

with rank 290 out of 5371 final valid motifs and a *z*-score of 22.61. As a direct comparison, identical results were reported by EXMOTIF.

## Conclusions and discussion

In this article, we introduced MoTeX-II, a word-based HPC tool for both single and structured MoTif eXtraction from large-scale datasets. A valid structured motif is called strictly valid if it occurs exactly, at least once, in any of the input sequences. By making this stricter assumption for motif validity, we showed how the structured motif extraction problem can be reduced to the fixed-length approximate string matching problem. Surprisingly, this natural and simple reduction has never been considered in the literature.

As a direct result of this reduction, and assuming that the length of every single motif is less than or equal to the size of the computer word, the runtime of MoTeX-II does not depend on (i) the length for motifs, (ii) the size of the alphabet, or (iii) the error thresholds. Moreover, MoTeX-II is guaranteed to find globally optimal solutions. It can identify structured motifs under the edit distance model or the Hamming distance model. Finally, MoTeX-II also comes in two HPC flavors: the OpenMP-based version and the MPI-based version.

State-of-the-art word-based motif extractors produce globally optimal solutions but exhibit many disadvantages. We demonstrated that MoTeX-II can alleviate these shortcomings for structured motif extraction from small-, medium-, and large-scale datasets. The scalability of our approach is due to the fact that the proposed algorithm is independent of the aforementioned input parameters and is highly parallelisable. For instance, we showed how the quadratic time complexity of MoTeX-II can be slashed, in theory and in practice, by using parallel computations; whereas suffix-tree-based motif extractors are difficult to parallelise effectively. The extensive experimental results presented are promising, both in terms of accuracy under statistical measures of significance as well as efficiency; a fact that suggests that further maintenance and development of MoTeX-II is desirable.

For future work, we will explore the possibility of optimising our approach by using lossless filters (see [19] and [20], for instance) for eliminating a possibly large fraction of the input that is guaranteed not to contain any valid occurrence before completing the motif inference task. Our main goal is to accurately detect single and structured motifs over massive sets of biological sequences representing a set of species. We are especially interested in discovering transcription factor binding sites whose conservation is decreasing as the evolutionary distance between those species increases. We plan to employ MoTeX-II in a phylogenetic framework to incorporate evolutionary information in the motif extraction process.

## Availability and requirements

• **Project name:**MoTeX

• **Project home page:**http://www.inf.kcl.ac.uk/research/projects/motex/

• **Operating system:** GNU/Linux

• **Programming language:** C

• **Other requirements:**gcc version 4.6.3 or higher

• **License:** GNU GPL

• **Any restrictions to use by non-academics:** licence needed

## Competing interests

The author declares that he has no competing interests.

## References

[B1] Lothaire M Applied Combinatorics on Words 2005 Cambridge, UK: Cambridge University Press

[B2] Pavesi G Mereghetti P Mauri G Pesole G **Weeder web: discovery of transcription factor binding sites in a set of sequences from co-regulated genes** Nucleic Acids Res 2004 32 Web-Server-Issue 199 203 10.1093/nar/gkh465 PMC441603 15215380

[B3] Rombauts S Déhais P Van Montagu M Rouzé P **PlantCARE, a plant cis-acting regulatory element database** Nucleic Acids Res 1999 27 1 295 296 10.1093/nar/27.1.295 9847207 PMC148162

[B4] van Helden J Andre B Vides CJ **Extracting regulatory sites from the upstream region of yeast genes by computational analysis of oligonucleotide frequencies** J Mol Biol 1998 281 5 827 842 10.1006/jmbi.1998.1947 9719638

[B5] Eskin E Pevzner PA **Finding composite regulatory patterns in dna sequences** Bioinformatics 2002 18 Suppl 1 354 363 10.1093/bioinformatics/18.suppl_1.S354 12169566

[B6] Sagot M-F **Spelling approximate repeated or common motifs using a suffix tree** Proceedings of the 3rd Latin American Symposium on Theoretical Informatics (LATIN’98) 1998 London, UK: Springer 374 390

[B7] Carvalho AM Freitas AT Oliveira AL Sagot M-F **An efficient algorithm for the identification of structured motifs in DNA promoter sequences** IEEE/ACM Trans Comput Biol Bioinformatics 2006 3 2 126 140 10.1109/TCBB.2006.16 17048399

[B8] Das M Dai HK **A survey of DNA motif finding algorithms** BMC Bioinformatics 2007 8 Suppl 7 21 10.1186/1471-2105-8-S7-S21 18047721 PMC2099490

[B9] Sinha S Tompa M **YMF: A program for discovery of novel transcription factor binding sites by statistical overrepresentation** Nucleic Acids Res 2003 31 13 3586 3588 10.1093/nar/gkg618 12824371 PMC169024

[B10] Floratou A Tata S Patel JM **Efficient and accurate discovery of patterns in sequence data sets** Knowl Data Eng IEEE Trans 2011 23 8 1154 1168

[B11] Pissis SP Stamatakis A Pavlidis P ACM **MoTeX: A word-based HPC tool for MoTif eXtraction** Fourth ACM International Conference on Bioinformatics and Computational Biology (ACM-BCB 2013) 2013 New York, NY, USA: ACM 13 22

[B12] Carvalho AM Marsan L Pisanti N Sagot M-F **RISOTTO: fast extraction of motifs with mismatches** Proceedings of the 7th Latin American Symposium on Theoretical Informatics (LATIN’06), Valdivia, Chile 2006 Berlin Heidelberg: Springer 757 768

[B13] Carvalho AM Freitas AT Oliveira AL Sagot M-F Chen Y-PP, Wong L **A highly scalable algorithm for the extraction of cis-regulatory regions** Proceedings of the 3rd Asia Pacific Bioinformatics Conference Advances in Bioinformatics and Computational Biology 2005 Singapore: Imperial College Press 273 282

[B14] Zhang Y Zaki M **EXMOTIF: efficient structured motif extraction** Algo Mol Biol 2006 1 1 1 18 10.1186/1748-7188-1-1 PMC1698483 17109757

[B15] Na JC Apostolico A Iliopoulos CS Park K **Truncated suffix trees and their application to data compression.** Theor Comput Sci 2003 304 1-3 87 101 10.1016/S0304-3975(03)00053-7

[B16] Jia C Carson M Yu J **A fast weak motif-finding algorithm based on community detection in graphs** BMC Bioinformatics 2013 14 1 1 14 10.1186/1471-2105-14-1 23865838 PMC3726413

[B17] Carvalho AM Oliveira AL Freitas AT Sagot M-F **A parallel algorithm for the extraction of structured motifs** Proceedings of the 2004 ACM Symposium on Applied Computing. SAC ’04 2004 Nicosia, Cyprus: ACM 147 153

[B18] Iliopoulos C Mouchard L Pinzon Y Brodal G, Frigioni D, Marchetti-Spaccamela A **The, Max-Shift algorithm for approximate string matching** Proceedings of the Fifth International Workshop on Algorithm Engineering (WAE 2001). Lecture Notes in Computer Science 2001 Denmark: Springer 13 25

[B19] Federico M Peterlongo P Pisanti N Sagot M-F Holub J, žďárek J **Finding long and multiple repeats with edit distance** Proceedings of the Prague Stringology Conference 2011 2011 Czech Republic: Czech Technical University in Prague 83 97

[B20] Federico M Peterlongo P Pisanti N Sagot M-F **RIME: Repeat identification** Discrete Appl Math 2014 163 Part 3 0 275 286

[B21] Crochemore M Hancart C Lecroq T Algorithms on Strings. New York 2007 USA: Cambridge University Press

[B22] Crochemore M Iliopoulos CS Pissis SP Elomaa T, Mannila H, Orponen P **A parallel algorithm for fixed-length approximate string-matching with**** *k* ****-mismatches** Algorithms and Applications. Lecture Notes in Computer Science 2010 Berlin Heidelberg: Springer 92 101

[B23] Marsan L Sagot M-F **Algorithms for extracting structured motifs using a suffix tree with an application to promoter and regulatory site consensus identification** J Comput Biol: J Comput Mol Cell Biol 2000 7 3-4 345 362 10.1089/106652700750050826 11108467

[B24] Buhler J Tompa M **Finding motifs using random projections** J Comput Biology: J Comput Mol Cell Biol 2002 9 2 225 242 10.1089/10665270252935430 12015879

[B25] **GenBank** [http://www.ncbi.nlm.nih.gov/genbank/]

[B26] **Ensembl Genome Browser** [http://www.ensembl.org/index.html]

[B27] Helden Jv Rios AF **Collado-Vides J: Discovering regulatory elements in non-coding sequences by analysis of spaced dyads** Nucleic Acids Res 2000 28 8 1808 1818 10.1093/nar/28.8.1808 10734201 PMC102821

[B28] GuhaThakurta D Stormo GD **Identifying target sites for cooperatively binding factors** Bioinformatics 2001 17 7 608 621 10.1093/bioinformatics/17.7.608 11448879

[B29] Zhu J Zhang MQ **SCPD: A promoter database of the Yeast Saccharomyces cerevisiae** Bioinformatics 1999 15 7 607 611 10.1093/bioinformatics/15.7.607 10487868

